# State of Menstrual Health Education in 2024: Content Analysis of US K‐12 Public School Education Standards

**DOI:** 10.1111/josh.70064

**Published:** 2025-08-12

**Authors:** Emily Richardson, Page D. Dobbs, Sarah Bemis, Hope Ballentine, Shristi Bhochhibhoya, Victor Kwaku Akakpo, Kylie Lovett

**Affiliations:** ^1^ Eleanor Mann School of Nursing, Department of Health, Human Performance and Recreation, Center for Public Health and Technology University of Arkansas Fayetteville Arkansas USA; ^2^ Associate Professor of Public Health, Department of Health, Human Performance and Recreation, Center for Public Health and Technology University of Arkansas Fayetteville Arkansas USA; ^3^ Heartland Whole Health Institute Bentonville Arkansas USA; ^4^ Eleanor Mann School of Nursing, University of Arkansas Fayetteville Arkansas USA; ^5^ Department of Health, Human Performance and Recreation, Center for Public Health and Technology University of Arkansas Fayetteville Arkansas USA; ^6^ Public Policy Program University of Arkansas Fayetteville Arkansas USA; ^7^ Department of Health, Human Performance and Recreation University of Arkansas Fayetteville Arkansas USA

**Keywords:** content analysis, education standards, menstrual health education, period poverty, public school

## Abstract

**Background:**

Exploratory study aiming to assess the prevalence and time of initiation of menstrual health curriculum in US public school education standards by state.

**Methods:**

US public school Kindergarten‐12th grade education standards from each state's Department of Education website, including D.C. (*n* = 51), were searched for menstruation, menstrual, menses, menopause, period, menarche, reproduction, puberty, and growth and development.

**Results:**

25.5% (*n* = 13) of US education standards include menstruation. Of these, constructs of comprehensive menstrual health were recorded including abnormal menstruation (*n* = 6), menstrual hygiene (*n* = 6), menopause (*n* = 1), and stigma (*n* = 7). Education is initiated in 3rd–5th grade (*n* = 6) and 6th–8th grade (*n* = 7). One state required boys and girls to be taught separately. Parents opt‐out choice is allowed in (*n* = 6) and (*n* = 6) requires certified teachers.

**Policy Implications:**

Isolating menstrual education as an independent framework is needed to advocate for educational inclusion in US public schools.

**Conclusions:**

Menstrual health education is profoundly rare in US K‐12 public education standards, despite 50 million students being impacted. Furthermore, comprehensive menstrual education does not currently exist in US education standards.

Menstrual health is a symptom of underlying well‐being or pathology, with many defining menstruation as a vital sign [1]. Abnormal menstruation may be an early indicator of anatomical malformation, metabolic dysfunction, or bleeding disorders [1]. Yet, there is little research studying menstrual health literacy or education. One report shows females scoring below 50% on menstrual knowledge [2], and another shows 86% of menarche‐related questions from an online forum of teen females were seeking information and reported feelings of doubt or uncertainty [3]. All support the need for specific menstrual education.

Teaching anticipatory guidance prior to menarche is recommended starting at 7 years old, with the average age of menarche in the US at 11.9 years and 10% of females starting before 10 years old [1, 4]. Females who received education pre‐menarche report decreased fear, higher rates of self‐efficacy, and positive behavior change [5]. While those who were uneducated experienced increased shame, fear of death, guilt, or embarrassment [6]. School‐based menstrual concerns among high‐risk 15–19 year‐old females include low confidence, lack of cultural awareness with menstrual products, and anxiety managing menstruation [7]. In addition, there is more pressure for boys to be educated, with menstrual educators reporting only 30% of them teach males [8].

Isolating menstrual health as an independent framework is imperative to improve menstrual health literacy [9]. Without this, menstruation assumedly gets integrated into sex education, resulting in menstruation only being taught in reference to menstrual hygiene products or pregnancy prevention [9, 10]. Whereas in Mexico City, 47 public schools implemented a menstrual education program in 5th–6th grade females, which upon completion showed a 6% decrease in stress, a 23% increase in knowledge, a 22% increase in identifying a trusted adult, and a 26% increase in learning about puberty in school [11]. This study supports the use of standard curriculums in public schools, and menstrual education increases knowledge and confidence while decreasing stress.

To achieve comprehensive menstrual health, one must have access to menstrual products and the ability to manage them; access to medical care for menstrual concerns; access to menstrual education; an environment free from stigma; and freedom to be an active member of society without menstrual limitations [10]. While 97% of parents support puberty education in high school [12], that is well beyond pre‐menarche, where education is most beneficial [1]. In addition, students in a qualitative study report their sexual reproductive health education was awkward, uninformative, or unprofessional, with the majority taught by coaches [13]. Conversely, even among a group of elementary school nurses specifically trained to teach menstrual health and hygiene, 53% requested more education on how to teach [8]. Therefore, the goal of this study is to determine what states have menstrual health specific education standards and to evaluate the content and facilitation of menstrual education in US public schools.

## 1 Methods

### 1.1 Participants and Procedures

This exploratory study used the six phases of thematic analysis to create a codebook using K‐12 health education standards in US public schools [14]. These were downloaded from their respective Department of Education's website July 15–17, 2024. If specific bills were mentioned within these standards, they were accessed and reviewed. Initially, one state from each of the four US geographic locations and DC were selected for the pilot study [15]. These included Georgia, Nevada, North Dakota, and Washington. A similar study used various sociodemographic metrics, 2022 voting patterns, and comprehensive sex education for pilot selection, with full justifications published elsewhere [16]. Inductive coding was used by the primary author to develop a comprehensive codebook with examples. Then, deductive coding was used by comparing found content to menstrual health standards as defined by D.C. Act 24–294, the mnemonic MENSES, National Sex Education Standards (NSES), and Global Menstrual Collective guidelines to ensure all recommended aspects of comprehensive menstrual education were included in the codebook.

Once the codebook was complete, two additional coders were trained and proceeded with a content analysis of each state's education standards (*n* = 51) with the primary author [17]. After five states were coded, Cohen's kappa light (*k = 0*.718) was calculated using IBM SPSS (version 29) showing sufficient interrater reliability [18]. Upon completion, all discrepancies were adjudicated by the primary author.

### 1.2 Instrumentation

Several standards were consulted to ensure all aspects of comprehensive menstrual education were assessed for including MENSES [19], NSES [20], and the Global Menstrual Collective [10]. MENSES is a healthcare assessment tool for comprehensive menstrual evaluations [19]. Only NSES items impacting menstrual literacy were considered and were all categorized under Anatomy and Physiology or Puberty and Adolescent Sexual Development [20]. The Global Menstrual Collective defined comprehensive menstrual health, but it is not standardized across literature yet [10]. In addition, DC's menstrual education standards served as an initial model policy [21]. Finally, grades where standards are taught were reported using comprehensive sex education (CSE) grade bands, K‐2nd grade, 3rd–5th grade, 6th–8th grade, 9th–12th grade [20]. However, some states cluster grade bands differently, such as 9th–12th, as high school curriculum, so initial grades were coded.

### 1.3 Data Analysis

Data was analyzed using IBM SPSS (version 29). Frequencies were calculated among the variables to identify which standards include menstrual‐specific education, reproductive hormones and anatomy, puberty, and growth and development. The codebook specified standards coded as growth and development must use this term in reference to puberty or reproduction for inclusion, acknowledging this is a general term and was often used in reference to nutrition (see Table 1). Once states that contain menstrual health education were analyzed, the recommendations from the Global Menstrual Collective were consulted to assess for comprehensive menstrual health [10]. Including education specific to menstruation, abnormal menstruation, menstrual hygiene, and menopause all within a stigma‐free environment (see Table 2). The next round of analysis documented what grade menstruation education was first introduced. If this was in a grade band, then the first grade was documented, unless otherwise specified. Of note, Kentucky's education standard states that menstrual education begins in the 5th grade, yet a pop‐up warning window comes up on the website that states according to Senate Bill 150 standard 5.1.6 concerning reproduction and puberty cannot be taught in or before 5th grade [22]. Additionally, standards were evaluated for their inclusion of required teacher training, gender of students taught, and parents' choice options (see Table 3).

**TABLE 1:** US K‐12 public school menstrual health education standards as of september 2024.

State	Menstruation	Reproductive hormones	Reproductive anatomy	Puberty	Growth and development
Alabama				✓	✓
Alaska					
Arizona					
Arkansas		✓	✓^a^, ^b^	✓	✓
California		✓	✓^a^ ^–^ ^c^	✓	✓
Colorado	✓	✓	✓^a^, ^b^		✓
Connecticut	✓	✓	✓^a^ ^–^ ^c^	✓	✓
Delaware					
District of Columbia	✓		✓^a^ ^–^ ^c^	✓	✓
Florida			✓	✓	✓
Georgia			✓^a^	✓	✓
Hawaii				✓	✓
Idaho				✓	✓
Illinois					
Indiana					
Iowa					
Kansas			✓^a^, ^b^	✓	✓
Kentucky	✓		✓^a^, ^b^	✓	✓
Louisiana	✓	✓	✓^a^, ^b^	✓	
Maine					
Maryland	✓		✓^a^ ^–^ ^c^	✓	✓
Massachusetts	✓	✓	✓^a^ ^–^ ^c^	✓	✓
Michigan	✓	✓	✓^a^ ^–^ ^c^	✓	✓
Minnesota					
Mississippi				✓	
Missouri	✓	✓	✓^a^ ^–^ ^c^	✓	✓
Montana			✓^a^, ^b^		✓
Nebraska					
Nevada		✓	✓^a^ ^–^ ^c^	✓	
New Hampshire				✓	✓
New Jersey		✓	✓^a^ ^–^ ^c^	✓	✓
New Mexico			✓^a^, ^b^	✓	
New York		✓	✓	✓	✓
North Carolina		✓	✓^a^, ^b^	✓	
North Dakota			✓^a^, ^b^	✓	✓
Ohio					
Oklahoma					
Oregon	✓✓	✓	✓^a^ ^–^ ^c^	✓	✓
Pennsylvania			✓^a^, ^b^		✓
Rhode Island			✓^a^, ^b^	✓	✓
South Carolina		✓	✓^a^, ^b^	✓	✓
South Dakota					
Tennessee	✓		✓^a^, ^b^	✓	✓
Texas	✓	✓	✓^a^, ^b^	✓	✓
Utah		✓	✓^a^, ^b^	✓	
Vermont			✓^a^, ^b^	✓	✓
Virginia	✓	✓	✓^a^, ^b^	✓	
Washington		✓	✓^a^ ^–^ ^c^	✓	✓
West Virginia			✓^a^ ^–^ ^c^	✓	
Wisconsin					
Wyoming					
Total *N* (%)	13 (25.5)	18 (35.3)	32 (62.7)	34 (66.7)	29 (56.9)

*Note*: ✓✓ Period Poverty is specified in education standards (*n =* 1, 1.9%).

^a^ Includes structures of reproductive anatomy (*n* = 30, 58.8%).

^b^ Includes function of reproductive anatomy (*n* = 29, 56.8%).

^c^ Anatomically correct names taught for reproductive anatomy (*n* = 12, 23.5%).

**TABLE 2:** State education standards specific to comprehensive menstrual health education.

	Menstruation	Abnormal menstruation	Menstrual hygiene	Menopause	Stigma‐free
California			✓^a^		
Colorado	✓		✓		
Connecticut	✓				
District of Columbia	✓	✓	✓	✓	✓
Kentucky	✓				✓
Louisiana	✓				✓
Maryland	✓				✓
Massachusetts	✓	✓	✓		✓
Michigan	✓		✓		✓
Missouri	✓	✓			
New Jersey			✓^a^		
Oregon	✓	✓	✓		✓
Tennessee	✓		✓		
Texas	✓	✓			
Virginia	✓	✓			
Total *N*	13	6	8	1	7

^a^ Does not specifically teach menstruation, but mentions menstrual hygiene products.

**TABLE 3:** Menstrual education course facilitation guidelines.

	Initial grade	Genders taught	Teacher training	Parent choice
Oregon	3rd*	—	CE	Opt‐out
District of Columbia	4th*	Both	—	Opt‐out
Michigan	4th*	—	CE	Opt‐out
Missouri	4th*	—	—	—
Texas	4th*	Separated	CE	Opt‐out
Colorado	5th	—	—	—
Connecticut	6th*	—	Certification	Mandatory
Louisiana	6th*	—	—	—
Maryland	6th*	—	CE	Mandatory
Massachusetts	6th*	—	CE	Opt‐out
Kentucky	6th**	—	—	Opt‐out
Tennessee	8th	—	—	—
Virginia	8th*	—	—	—

*Note*: Initial grade references the first grade where menstruation is a standard. If this is a grade band, the first grade in the band is listed.

Abbreviations: CE, Continuing education or professional development; —, Non‐specific.

* Taught in more than one grade.

** KY education standard is 5th grade, but pop‐up warning denotes it cannot be included in 5th grade or before.

## 2 Results

### 2.1 Menstrual Education Standards

Initially, all standards were coded for inclusion of menstrual education. Menstruation (*n* = 13, 25.5%) was only coded yes if the word menstrual, menses, menarche, or menstruation was specifically referenced. To ensure that all menstrual concepts were considered, coders searched for reproductive anatomy (*n* = 32, 62.7%) structures (*n* = 30, 58.8%) and function (*n* = 29, 56.8%), reproductive hormones (*n* = 18, 35.3%), puberty (*n* = 34, 66.7%), and growth and development (*n* = 29, 56.9%). Anatomically correct terms for reproductive anatomy are specified in 12 (23.5%) standards. See Table 1 for state specifics.

#### 2.1.1 Comprehensive Menstrual Health Standards

While 29.4% (*n* = 15) of standards include menstrual health, *n* = 13 (25.5%) have education standards specific to menstruation or the menstrual cycle. Abnormalities in menstruation are included in *n* = 6 (11.8%) standards; examples include dysmenorrhea, abnormal bleeding, and pre‐menstrual syndrome. Menstrual hygiene is in *n* = 8 (15.7%) standards, which includes education for menstrual products and personal care specific to menstruation. Menopause is lacking in every standard except *n* = 1 (2%). However, the scope of menopause in DC's standard is limited, only referencing it as an example of menstrual change. Recommendations that education be made in an inclusive, safe, or stigma‐free environment are found in *n* = 7 (13.7%) standards.

### 2.2 Menstrual Education Course Facilitation Guidelines

Oregon is the only state initiating menstrual education in 3rd grade, reaching 8–9 year‐olds. States initiating in 4th–5th grades were 13.7% (*n* = 7). Another 13.7% (*n* = 7) were in the 6th–8th band, and New Jersey (*n* = 1, 2%) does not specifically teach menstruation but teaches menstrual health products starting in 9th grade, usually 14–15 year‐olds. Therefore, most females have been menstruating for at least 3 years before receiving education [4]. Those specifying classroom gender composition were 3.9% (*n* = 2); DC requires males and females to receive menstrual education and Texas mandates genders be separated. For educators, 9.8% (*n* = 5) require content‐specific professional development or continuing education, 2% (*n* = 1) require additional certification, and 17.6% (*n* = 9) did not specify who is teaching the content or if they require content‐specific education. Finally, 11.8% (*n* = 6) of the standards allow parents to review the content prior to their child, and they can opt‐out. Opt‐out allows parents to exempt their child from receiving the education without penalty. Standards mandating reproductive health education are included in 3.9% (*n* = 2). No reports of parent choice exist in 13.7% (*n* = 7) of standards. However, common practices may exist by district.

## 3 Discussion

**FIGURE 1:**
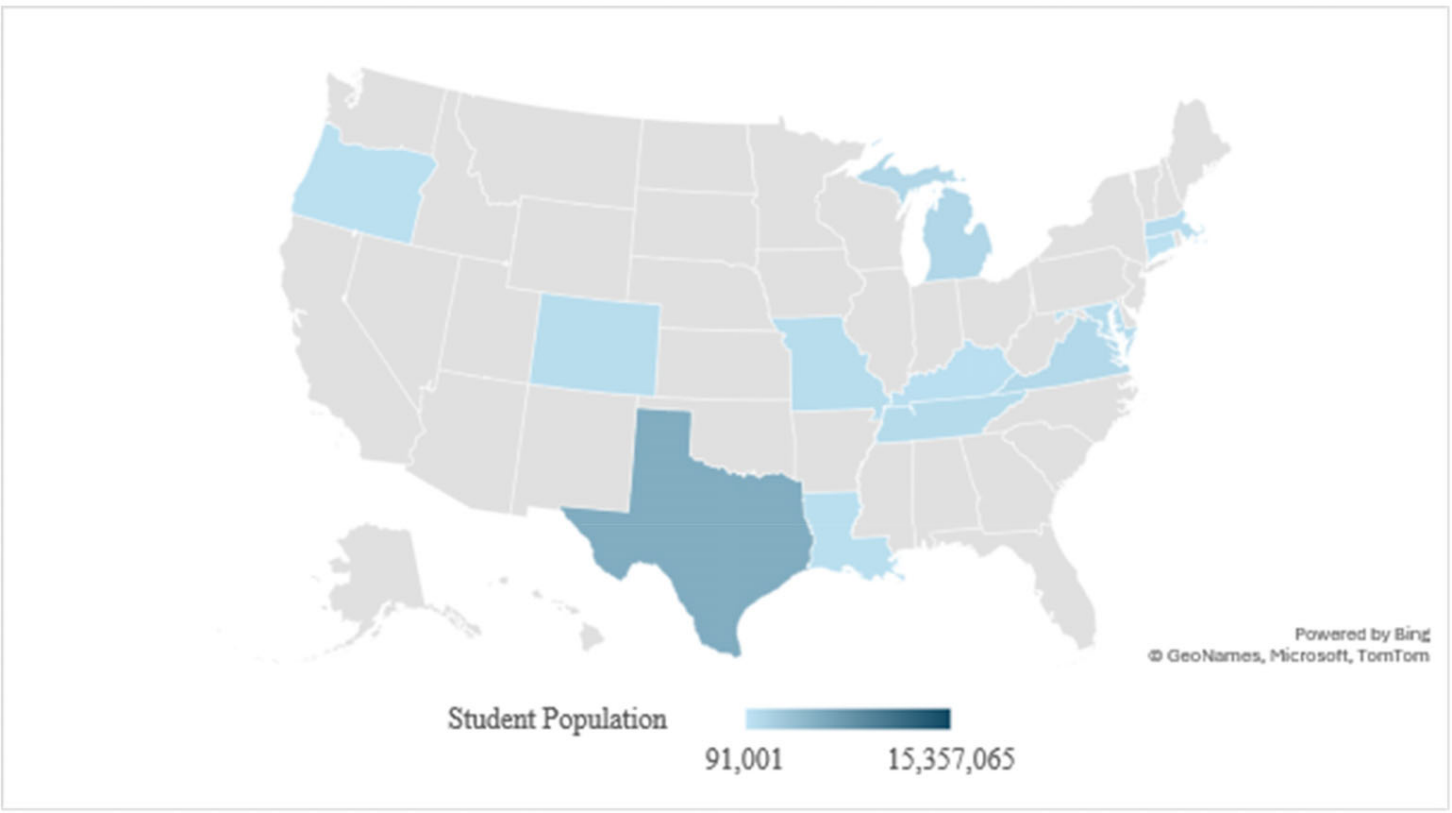
US population of K‐12th grade students in 2022 enrolled in states with menstrual education standards.

In 2022 there were 49 618 464 students enrolled in US public schools in grades K–12th grade [23]. With approximately 50 million students enrolled, only 15 357 065 of those attend public schools in one of the 13 states with menstrual health education standards [23]. See Figure 1 for a range of student populations receiving menstrual education by state. This leaves approximately 70% of US public school students receiving education in states that do not mandate menstrual education. This is a profound portion of the student population and can have a negative impact on student learning [7].

Increasing menstrual health literacy decreases absenteeism related to menstrual disorders, improves perceived overall well‐being, and menstrual health awareness, showing that increased knowledge can impact behavior [24]. Notably, many teachers are supportive of mandating curriculum standards and voice concerns over the normalization of high‐risk behaviors and the spread of misinformation when students seek education from the internet in the absence of a trusted adult [25]. However, as evident from these results, menstrual education is often incomplete or unspecified in K‐12 US public education standards [9, 26]. Based on comprehensive menstrual health frameworks, there is not a single state whose education standards fully meet these requirements [10, 19, 20].

To achieve comprehensive menstrual health, all who menstruate should have access to information on how to manage their menstrual cycle, healthy hygiene practices, developmentally appropriate information about menstruation, menstrual changes over the lifespan, and education differentiating normal and abnormal menstruation [10]. Yet, comprehensive menstrual health education for both genders is such a novel idea, DC made national headlines when they became the first to implement universal menstrual health education standards in the 2023–2024 school year [27]. Not only do these results reveal a lack of menstrual education, but they expose a further need for comprehensive menstrual education standards.

## 4 Policy Implications

These results show a significant lack in menstrual health standards across US public education, leaving the vast majority of students uneducated. Initially, menstrual education should be adopted as an independent framework allowing policymakers, healthcare providers, and educators to develop standard comprehensive menstrual education curriculum apart from sex education. Additionally, advocacy for pre‐menarche education implementation is crucial for positive outcomes and the reduction of fear and stigma.

## 5 Limitations

History bias may cause an internal threat due to the ever‐changing education policies at the state level. To overcome this, exact dates for which findings can be generalized are listed. Participant selection for the pilot study may also impact the initial codebook, with potential themes missed. To overcome this, discussions occurred between coders throughout the process to ensure no content was uncoded. Finally, the lack of an independent menstrual health framework may have increased information access barriers [10]. Furthermore, discrepancies between policy and teaching styles or values can exist, making it impossible to know what is being taught in individual classrooms [28].

## 6 Conclusions

Until menstrual education is isolated as its own standard, it will continue to get caught in sex education debates; therefore, to ensure menstrual education is initiated pre‐menarche, it is imperative this differentiation occurs [29, 30]. Withholding education contributes to social withdrawal, decreased school participation, and frustration with school policies around managing dysmenorrhea or curriculum [7]. Furthermore, the lack of teacher training cannot be dismissed. Among those certified to teach puberty in secondary schools, 67% report being “very confident” and 29% “confident,” yet 90% request additional training to build knowledge and student engagement strategies [31]. Menstrual health educators in the UK, where menstrual education is standard, also report feelings of embarrassment and not knowing how to teach boys, supporting the need for teacher training [32].

Access to menstrual products is a construct of comprehensive menstrual education with support that access barriers negatively impact school attendance and academic attainment [33]. While some states are already writing bills to combat period poverty [34], provision of products should be paired with education as a primary intervention. Coders assessed for period poverty or menstrual equity in education standards, with Oregon being the only state to reference provision of products; yet these bills may be located outside of education standards.

Future studies could analyze states' menstrual equity bills for education requirements. Additionally, studies should evaluate the effects of early menstrual education on fear, stigma, double standard ideology, and bias for both genders. Also, they may evaluate teacher self‐efficacy and menstrual health knowledge. Finally, many education standards report parents as primarily responsible for teaching children sexual reproductive health; thus, studies should evaluate parent competencies and intentions to teach menstrual education, or attitudes and beliefs about pre‐pubescent children receiving menstrual education in schools.

## Ethics Statement

Preparation of this paper did not involve primary research or data collection involving human subjects, and therefore, no institutional review board examination or approval was required.

## Conflicts of Interest

The authors declare no conflicts of interest.
